# Can Psychodynamically Oriented Early Prevention for “Children-at-Risk” in Urban Areas With High Social Problem Density Strengthen Their Developmental Potential? A Cluster Randomized Trial of Two Kindergarten-Based Prevention Programs

**DOI:** 10.3389/fpsyg.2020.599477

**Published:** 2020-12-10

**Authors:** Tamara Fischmann, Lorena K. Asseburg, Jonathan Green, Felicitas Hug, Verena Neubert, Ming Wan, Marianne Leuzinger-Bohleber

**Affiliations:** ^1^International Psychoanalytic University Berlin, Berlin, Germany; ^2^Sigmund-Freud-Institut, Frankfurt am Main, Germany; ^3^The University of Manchester, Manchester, United Kingdom; ^4^University Medicine Johannes-Gutenberg-University, Mainz, Germany

**Keywords:** children at risk, prevention program evaluation, object attachment, randomized controlled trial, risk/protective factor, resilience

## Abstract

Children who live on the margins of society are disadvantaged in achieving their developmental potential because of the lack of a necessary stable environment and nurturing care. Many early prevention programs aim at mitigating such effects, but often the evaluation of their long-term effect is missing. The aim of the study presented here was to evaluate such long-term effects in two prevention programs for children-at-risk growing up in deprived social environments focusing on child attachment representation as the primary outcome as well as on self-reflective capacities of teachers taking care of these children. The latter was a key component for promoting resilient behavior in children. Five hundred and twenty-six children aged 36 to 60 months at risk due to immigration status, low family socio-economic status and child behavior were examined in a cluster-randomized study comparing two preventions, the psychodynamic, attachment-based holistic approach EARLY STEPS (ES) with the classroom based FAUSTLOS (FA) for their efficacy. Primary outcome was the child attachment representation measured by the Manchester Child Attachment Story Task (MCAST). Secondary outcomes were derived from (a) the Caregiver-Teacher Report Form (C-TRF: problem behaviors, including anxiety/depressive symptoms, emotional-reactive and somatic problems, social withdrawal, aggressive behavior, and attention deficit), from (b) the Strength and Difficulties Questionnaire (SDQ, parent version: resilience and wellbeing) and (c) Self-Reflective Scales for teachers (SRS: self-reflective capacities of teachers). Compared to baseline, attachment and behavioral problems improved in both programs. ES led to more secure and more organized attachment representations (medium effect sizes). Aggressive behavior and externalizing problems were reduced in the FA group compared with ES, particularly in boys (medium effect sizes). Self-reflective capacities of the teachers increased only in the ES group. High correlation between children’s attachment type with the number of social risk factors and the increase of problematic social behavior strongly indicate that an increase in teachers’ self-reflective capacities helps to change children’s attachment patterns which thus strengthens the resilience of these children-at-risk [An ethical vote from LPPKJP 2009-02-25 was obtained and the trial registered; Clinical trial registration information: The trial was registered 14.02.2012 (DRKS00003500; https://www.drks.de)].

## Introduction

Emotional neglect, experiences of violence, threat and traumatization have severe effects on psychological development as has been demonstrated impressively by many clinical, empirical and interdisciplinary studies (Bohleber, 2000; De Bellis and Thomas, 2003; Becker-Stoll et al., 2009; Leuzinger-Bohleber et al., 2017a, 2010, 2020). Findings from various longitudinal studies indicate that such children have a poor prognosis, exhibiting aggressive-destructive behavior and severe psychological problems and performing below-average at school (Green et al., 2000; Helmsen et al., 2012; Petermann and Petermann, 2012). In many studies it is discussed that the specific experience of a child as well as their family and social environment must be taken into account in order to understand the short and long-term consequences of early-life adversities in detail in order to respond to them in an adequate individual way. As will be discussed in the following, one of the strengths of a psychodynamic/psychoanalytic prevention approach is that it always tries to understand such individual characteristics of early childhood adversities and traumatization, which is seen as the presupposition for offering adequate prevention to the individual child and the family.

It is interesting that such individualized prevention strategies increasingly receive interdisciplinary support. To mention just one example: A meta-analysis by Colich et al. (2019) found that early-life adversity (ELA) involving threat (e.g., violence exposure) was associated with accelerated biological aging across multiple metrics, whereas exposure to deprivation (e.g., neglect, institutional rearing) and low socio-economic status (SES) was not. The authors meta-analyzed 46 studies (*n* = 64,925) the examining association of ELA with pubertal timing and cellular aging (telomere length and DNA methylation age) in order to understand the short and long-term consequences of early-life adversities in detail and to take corrective action in an individual way. The authors systematically reviewed 19 studies (*n* = 2,276) examining ELA and neural markers of accelerated development (cortical thickness and amygdala-prefrontal cortex functional connectivity) and evaluated whether associations of ELA with biological aging vary according to the nature of adversity experienced. ELA overall was associated with accelerated pubertal timing (*d* = 0.12) and cellular aging (*d* = 0.32). “Moderator analysis revealed that ELA characterized by threat (*d* = 0.26), but not deprivation or SES, was associated with accelerated pubertal development. Similarly, exposure to threat-related ELA was associated with accelerated cellular aging (*d* = 0.43), but not deprivation or SES. Systematic review revealed associations between ELA and accelerated cortical thinning, with threat. Related ELA was consistently associated with thinning in the ventromedial prefrontal cortex, and deprivation and SES was associated with thinning in frontoparietal, default, and visual networks. There was no consistent association of ELA with amygdala-PFC connectivity. These findings suggest specificity in the types of early environmental experiences associated with accelerated biological aging and highlight the importance of how accelerated aging contributes to health disparities and whether this process can be mitigated through early intervention” (Colich et al., 2019, p. 2).

As these studies show, accelerated biological aging is particularly impacted by exposure to violence. This corresponds to the findings of attachment research that exposure to domestic violence is one of the major factors for the development of a disorganized attachment (see following paragraph) and are a great risk for the development of the children. Therefore, early prevention efforts are particularly important for this group of children. But also children exposed to other early-life adversities have a poor prognosis, exhibiting aggressive-destructive behavior and severe psychological problems as well as performing below-average at school and thus need early additional support and help (Green et al., 2000; Hanson et al., 2011; Bøe et al., 2012; Helmsen et al., 2012; Petermann and Petermann, 2012).

This is why an emerging body of research proposes early prevention programs for children with low family income, migration background and other early-life adversities (Ludwig and Phillips, 2008; Leuzinger-Bohleber et al., 2013). Several studies of early prevention programs displayed an improvement in cognitive, emotional, and behavioral development and a decrease in delinquency and crime in adulthood (Nelson et al., 2003; Emde and Leuzinger-Bohleber(eds), 2014). Especially findings of research on attachment styles revealed a correlation between type of attachment and psychological and cognitive performance. In 1951, Bowlby identified infants’ biological need to form attachment to caregivers to build a secure base from which they can explore the world. According to Bowlby, human attachment behavior is biologically programmed and a means of survival, as it directs the infant to seek proximity to his/her caregiver when he/she needs protection from danger or emotional support. The infant’s attachment to his/her caregivers allows him/her to build trust and thereby explore his/her surroundings and assume social relationships (Bowlby, 1951). Ainsworth expanded on Bowlby’s seminal work and conducted empirical research on attachment which involved investigating the effects of early traumatization on attachment patterns and children’s subsequent development (Cassidy and Shaver, 2008). She found a strong correlation between neglect during early childhood and emotional problems during adolescence (Wolraich et al., 1996; Gunnar et al., 2000; Dozier et al., 2002; Teicher et al., 2002; De Bellis and Thomas, 2003; Cassidy and Shaver, 2008; Maheu et al., 2010; Rutherford and Mayes, 2014) which often led to anxiety disorders, depression, and even suicide (Pine, 2003, 2007). According to Ensink et al. (2017) not only secure attachment but also the acquirement of the ability of mentalization (symbolization) is crucial in terms of a healthy psychological development. Humans who are exposed to violence, threat or severe trauma show most likely a disturbed ability of mentalization and poor emotional processing. Fonagy and Allison (2014) linked studies from the field of attachment and mentalization research with studies on epistemic trust. Secure attachment and a well-developed capability to mentalize, which can also be seen in children with low SES and a migration background, lead to epistemic trust allowing e.g., children in the age of 5–6 years to trust their own perceptions in conflictual situations which subsequently is of course a great advantage for the cognitive, affective and social development (Correau and Rochette, 2009). Therefore, it is very important to offer early prevention to children-at risk particularly in environments with an increased risk for violence, threat and trauma, i.e., an early prevention offer that explores ways to prevent violence, that strengthens resilience of these children, that encourages prosocial behavior, and that supports social integration.

Psychoanalysts are especially knowledgeable on early neglect, domestic violence and traumatization due to their clinical experience with such patients as well as due to numerous developmental studies (see e.g., Stern, 1985; Emde and Leuzinger-Bohleber(eds), 2014). Adopting the insight of mostly US-based prevention studies the Sigmund-Freud-Institut (SFI) repeatedly conducted early prevention studies in cooperation with the institute for Analytical Child and Adolescence Psychotherapy (VAKJP) and the Centre for Research on individual Development and Adaptive Education of children at risk (IDeA) of the federal funding initiative for excellence in Hesse (LOEWE). One such study, exploring the effects of early prevention – the representative Frankfurt Prevention Study (FP) carried out in 14 daycare centers in Frankfurt from 2003 to 2006 with 4,500 children from low-SES, middle-SES and high-SES backgrounds showed, that aggressive (*p* = 0.02) and anxious (*p* = 0.03) behavior of boys and girls and hyperactivity (*p* = 0.001) in girls-only was reduced significantly through implementation of a psychoanalytically based early prevention program (Leuzinger-Bohleber, 2007; Leuzinger-Bohleber et al., 2016). These results encouraged the research group to replicate this study with a specific focus on the differential effects of two established prevention programs – *Early Steps* (ES; Leuzinger-Bohleber, 2010; Leuzinger-Bohleber and Fischmann, 2010; Leuzinger-Bohleber et al., 2010) and *Faustlos* (FA; German version of Second Step; Cierpka, 2004; Schick and Cierpka, 2006) in a population of children-at-risk (EVA study) in a cluster randomized controlled trial (CRCT) based on criteria of SES, Aggression, Anxiety and Hyperactivity. Considering one of the major findings of the Head Start program (Emde, 2014), the main hypotheses of the EVA study is that for children at great developmental risk (i.e., with a disorganized attachment pattern) the elaborated, individualized and psychoanalytically oriented ES program will show long-lasting positive effects on children’s social behavior and enable them to achieve more secure attachment behavior than in the standardized, not individualized FA program.

The *Early Steps* program integrates research results from both attachment theory and psychoanalytic development and prevention research (see e.g., Emde and Leuzinger-Bohleber(eds), 2014; Leuzinger-Bohleber, 2015) focusing on change of children’s Inner Working Models (IWM) from an insecure IWM exhibiting insecure attachment representations toward safe IWMs with secure attachment representations.

The EVA project was strongly influenced by the well-known large prevention program, the Early Head Start community-based program for low-income families (Elicker et al., 2013), which links families with needed services such as medical, mental health, nutrition, and education and additionally provides a place for children to experience consistent, nurturing relationships and stable, ongoing routines (Love, 2010). An Early Head Start evaluation showed that caregiver–child relationships were generally positive in terms of attachment security after fostering a supportive, relationship between mother and program staff (Love et al., 2005).

Thus, it seems that secure attachment and a well-developed capability to mentalize may lead to an ‘epistemic trust’ (Asseburg, previously Hartmann et al., 2015; Bevington et al., 2017; Schwarzer, 2018; Dlugosch and Henter, 2020) and to confer advantages in cognitive, affective and social development as was hypothesized by Fonagy and Allison (2014). In contrast, children with disorganized attachment representations due to neglect, exposure to violence and early traumatic experiences have a poor prognosis, exhibiting aggressive-destructive behavior and severe psychological and emotional problems (De Bellis and Thomas, 2003; Fearon et al., 2010; Groh et al., 2012, 2017; Helmsen et al., 2012; Cassidy and Shaver, 2016; Facompré et al., 2017; Thomason and Marusak, 2017).

Therefore, the following have been the central research questions of the EVA study:

(1) Will the psychodynamic (attachment) based, holistic *Early Steps* program be superior to the classroom based *Faustlos* program in promoting child attachment representations?
(2) Will the *Faustlos* program be more effective in improving social behavior and adaptation than *Early Steps* but not change the attachment representations?^1^

In order to answer these research questions with sufficient external validity, the interdisciplinary research team chose a combination of a naturalistic and an experimental design, despite the fact that a pure experimental design for testing the efficacy of prevention programs according to the criteria of evidence-based medicine is preferred by most researchers because of the high internal validity that such a procedure offers. However, such studies often fail to achieve external validity. Therefore, their results can often hardly be transferred to practice. In contrast, naturalistic studies often have a high external validity, but have to accept some fuzziness in the way intervention programs are implemented in the project.

The interdisciplinary research group of the EVA study, in collaboration with one of the leading statisticians, Prof. Dr. Bernhard Rüger (Institute for Statistics of the University of Munich), tried to find an innovative solution in dealing with the specific challenges of studies in this area by combining naturalistic and experimental components in the design of the EVA study: The research team chose to use the existing professional resources for the promotion of verifiably needy at-risk children in public Kindergartens in the city of Frankfurt (hence “naturalistic study”) on the one hand and on the other applied the highest possible standards in the evaluation of the prevention offers (hence “experimental study”). The details of this design are described in the following sections.

## Materials and Methods

### Participants

Kindergarten children aged 3 to 6 years were recruited across 14 representative Kindergartens within areas with neighborhood deprivation, high migration background and low socio-economic status (SES). All cluster (Kindergarten) children were eligible to receive the interventions if they were aged 3 years or older at the beginning of the study (see Supplementary 1 and Supplementary Table S1 for clustering).

A short case example may illustrate the children-at-risk of the study.

Case example Ahmed:

Ahmed was to be excluded from the day-care center at the age of four because he had seriously injured another child’s head in one of his frequent conflicts with other children. According to the Manchester Child Attachment Story Task (MCAST) assessment Ahmed had a disorganized attachment representation. In the doll house scene in which the child loses its mother in the mall, the mother first hits the child, then the child kills the mother in a 15-min violent episode.

In the psychoanalytic supervision during these weeks, in which enormous feelings of violence, anger and aggression of many team members were in the center, it was decided to implement a “Round Table” with Ahmed’s mother and the social worker by the Youth Welfare Office who takes care of her, the responsible teacher and the head of the Kindergarten and the chair of the EVA project as participants. This meeting revealed that Ahmed’s mother was a severely traumatized (sexually abused) immigrant woman from Kurdistan, separated from her alcoholic husband 2 years ago. Ahmed had often witnessed extreme violence in the family.

As a result of the “Round Table,” the mother agreed to a psychodynamic child therapy to take place in the Kindergarten itself. Fortunately, Ahmed’s aggressive breakthroughs became less frequent after only a few months. He was able to establish a better emotional and social contact with the teachers and the other children.

In Ahmed’s Child Attachment Interview (after 2.5 years) a more organized attachment strategy could be seen, characterized by a dismissing attitude toward the attachment relationship at the age of seven. He now managed his behavior well, was academically engaged and had no social problems, but still without deeper friendships to other children.

### Trial Flow

**FIGURE 1:**
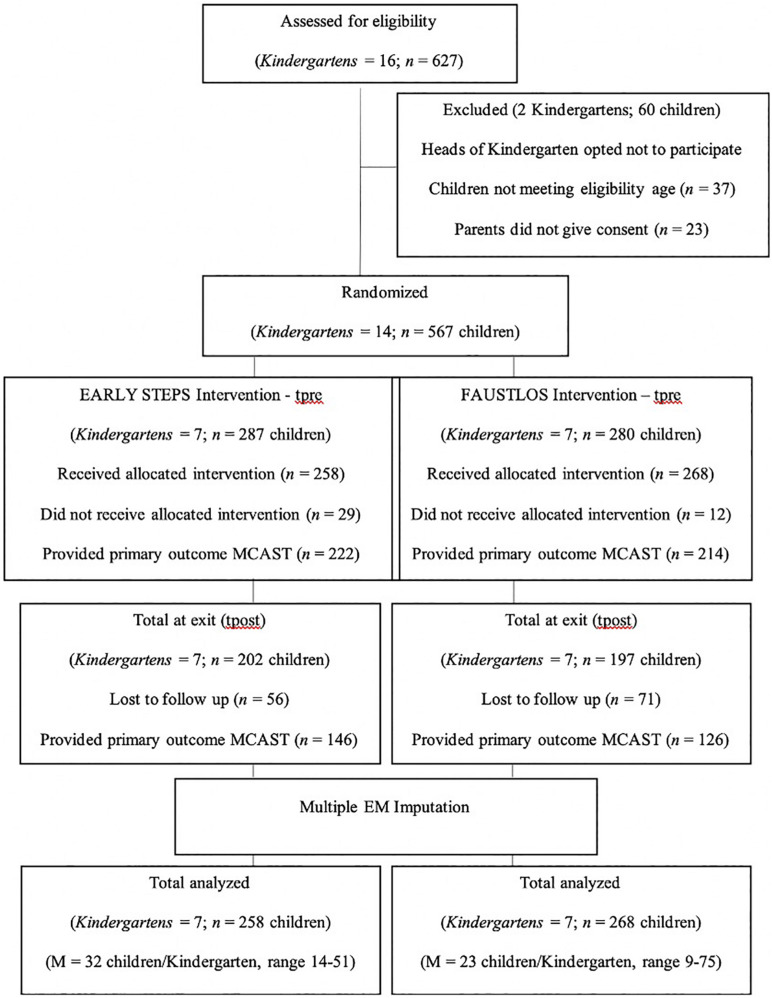
Consort chart.

A total of 16 Kindergartens were randomly picked and contacted and screened for eligibility (see Supplementary Table S1). Of those, two opted not to participate, leaving 14 eligible Kindergartens including 627 children. Of those, 14 Kindergartens totaling 567 children were included in the study after obtaining written consent from the parents. The consort chart (see Figure 1) shows the number of Kindergartens and participants included, reasons for exclusion, and the available main outcome assessments, MCAST, at baseline (prior to intervention – tpre) and 2 years post intervention (tpost), separately for each intervention arm.

### Randomization, Sample Size and Methods to Preserve Blindness

Since both prevention-offerings – *Early Steps* and *Faustlos* – were directed both at the individual child and at the entire group of children (and teachers), randomization was carried out at cluster level (Kindergartens). In addition, ethical considerations were taken into account in design and randomization: All children in this high-risk group had to be offered a form of prevention. An untreated control group would not have been ethically responsible.

#### Sample Size

Sample size was calculated with a method (Eldridge et al., 2006) that considers the intra-cluster correlation coefficient (ICC), the number of events, the expected effect and the power of the study by using the pre/post differences in outcome measures of the Frankfurt Prevention Study. This method yielded seven clusters (i.e., 1 Kindergarten = 1 cluster) with a mean cluster size of 20 (children). The sample size calculation and power analysis were based on α = 0.05 at a power of 0.80, with a yielded effect size of *d* = 0.5, using the pre/post differences of the FP study. As we applied a cluster randomized controlled trial design with the Kindergartens as given clusters, we used a formula for a conservative estimate of sample-size requirements for trials using cluster-level analyses weighted by cluster size. The formula^2^ for the corrected sample size consisted of the coefficient of variation (*CV*) for trials with unequal cluster sizes and ICC within the clusters and the mean cluster size *m*. Again, using the findings of the FP, the estimated coefficient of variation is *CV* = 0.467, and the estimated intra-class correlation coefficient (based on the pre/post differences including the *CV*) is *ICC* = 0.0465. We expected a mean cluster size *m* = 20 or at least *m* = 17 children. The corrected sample size for the first scenario would be *n*^∗^ = 131.44 (if *m* = 20) divided by 20 = 6.57; in the second scenario *n*^∗^ = 120.73 (if *m* = 17) divided by 17 *n*^∗^ = 7.10. For both scenarios, seven clusters—respectively, seven Kindergartens per treatment—should be selected. This sample size estimation indicated that 14 Kindergartens out of 114 Kindergartens of the Frankfurt municipality were needed. Paired units (Kindergartens) were randomly assigned (by TF at the principal investigator’s office at the Sigmund-Freud-Institut, using computer-created random numbers) to one of the intervention arms (see Trial Flow, Figure 1).

#### Data Protection

The intervention allocation for each Kindergarten was kept at the principal investigator’s office in Frankfurt throughout the study. When local assessors of outcome were ready to assess attachment security, they did not know of intervention allocation at any time point.

### Preventions

Since at the time of the EVA study the *Faustlos* prevention was offered in almost all public Kindergartens, this served as “prevention-as-usual” in the EVA study – analogous to designs in comparative psychotherapy research. As mentioned above, the research question of the EVA study was based on the results of the Frankfurt prevention study, which showed that although the *Faustlos* prevention certainly improved social behavior of many children, it did not prove to be sufficient and sustainable for particularly disadvantaged children. Therefore, the EVA study examined whether the development of these children-at-risk could be supported by an additional prevention offer.

A randomized cluster-controlled trial was conducted in which the *Early Steps* program including the *Faustlos* group program (ES condition) was compared to *Faustlos* only (FA condition). Both arms were 2 years in duration, with assessments collected pre- and post-intervention. The interventions pertained to the cluster level, i.e., Kindergartens.

#### Early Steps

The ES prevention offer is a multi-faceted individualized, psychodynamic holistic prevention program targeting attachment security in young children via an alternative, consistent and benevolent relationship experience. It consists of four modules (see also case example, above): (1) Psychodynamic 14-day case supervision for the teachers of young at-risk children, aiming to deepen teachers’ understanding of children’s unconscious conflicts and motives and thereby help them handle difficult conflicts and situations. Special focus in supervision lies on the interaction between child and teacher, including critical self-reflections of the teacher’s own emotional reactions, fantasies as well as possible counter-transference reactions. (2) Weekly presence of an experienced psychodynamic child and adolescent psychotherapist in the Kindergarten who offers counseling to parents and teachers. (3) These child therapists in the Kindergartens also offered psychotherapies to children who are specially in need (see case example above) (4) ES also includes FA (see below).

#### Faustlos

*Faustlos* was originally developed in the United States^3^, and is a classroom-based prevention program consisting of a standardized curriculum aimed at reducing impulsive and aggressive behavior and increasing socio-emotional competence in preschool-aged children (Jones et al., 2015). It is composed of three main parts: (1) development of empathy, (2) impulse control and (3) handling of conflicts and anger (Cierpka, 2004), and has shown effectiveness in improving socio-emotional competence (Jones et al., 2015). The program involves 28 teacher-led lessons to coach children in identifying with the feelings of other children, adopting their perspective, responding empathically, and using problem-solving skills to reduce impulsive or aggressive behavior.

### Study Design

### Primary and Secondary Outcome Measures

**FIGURE 2:**
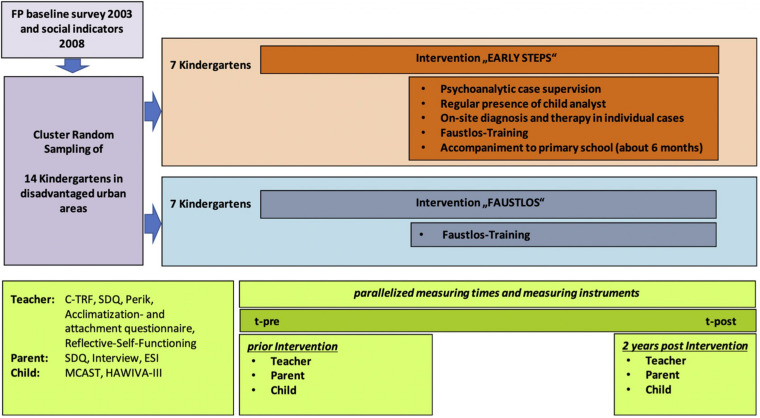
EVA design.

Primary outcome was the transformation of attachment representations of children-at-risk measured by the MCAST (see below). Secondary outcomes were problem behavior change in children directly correlated with the implemented interventions and measured by the C-TFR (see below), SDQ and Reflective Functioning (RF) of teachers (see Figure 2).

#### Primary Outcome Measure

*Manchester Child Attachment Story Task* (MCAST; Green et al., 2000): This validated semi-structured story stem completion task method for children aged 4 to 8 years incorporates dollhouse play to elicit the child’s internal representations concerning their attachment with a primary caregiver. The trained interviewer initiates four story vignettes of everyday scenarios involving doll figures that the child has selected to represent the child and caregiver and which are designed to activate attachment-relevant distress (e.g., the child doll wakes up in the middle of the night and has a bad dream; the child doll loses its mother in a mall). The child is then asked to complete the story with the use of non-leading prompts (e.g., “And then what happens?) and standard probe questions (e.g., “How is mom-doll feeling now?). The task is preceded with a control/practice vignette to ensure the child understands the task requirements, to allow the interviewer to build rapport with the child and to serve as a baseline of the child’s usual behavior and storytelling skill. The videotaped story completions are then coded by trained raters blinded to group allocation, measurement point and participant information, by rating the child’s behavioral, affective and verbal responses within the depicted doll play on a series of attachment-related rating scales according to the MCAST manual (see Green et al., 2000) to provide a final single measure of attachment classification (secure – B, insecure-avoidant – A, insecure-ambivalent – C or insecure-disorganized – D). The child’s story responses and behavior around attempts to respond are understood to reflect their internal representations of the attachment relationship, such as expectations about responsive care and how organized or fragile these representations are. The MCAST has been extensively used internationally with at-risk and clinical groups (Allen et al., 2018). The independent and blinded reliability test of twenty videos by five raters of EVA (concerning the attachment classification A vs. B vs. C vs. D) resulted in Fleiss’ Kappa > 0.62 (95% CI = 0.55–0.70).

#### Secondary Outcome Measure

*Caregiver-Teacher Form* 2 - 5 (C-TRF; Achenbach, 1997): This Kindergarten teacher-report measure assesses problem behavior regarding anxiousness/depressiveness, emotional-reactive and somatic problems, social retreat, aggressive behavior, attention deficit and other problems.

*Strengths and Difficulties Questionnaire* (SDQ; Goodman, 1997): This widely used 25-item measure presents statements about a child’s behavior for third-party assessment (parent) asking to complete how true each describes the child over the last month (not true/somewhat true/certainly true). The responses based on five areas of child problems/prosocial behavior generate five scales (emotional symptoms, conduct problems, hyperactivity-inattention, peer problems, and prosocial behavior) and the first four areas were summed to generate a child problems score for the current study. The validity and reliability of this score of the parent SDQ are satisfactory to detect psychosocial problems in children from multi-ethnic backgrounds (Mieloo et al., 2014).

*Interview for Pedagogues to assess their Reflexivity* (IRS; Asseburg, prev. Hartmann et al., 2015): This interview on teachers’ reflexivity is rated with the German version of *Reflective Functioning Scale* (RFS; Fonagy et al., 1998) yielding scores from −1 to 9 for low respectively high reflective functioning.

### Statistical Analysis

Data structure for the primary outcome included continuous categorical as well as nominal data, and therefore, Student’s *t*-tests and Wilcoxon tests were used for baseline comparison between treatment groups (to assess the success of randomization). We used cluster-specific methods because Kindergartens rather than children were randomized, and we expected that variance in how children’s attachment changed would be explained by the Kindergarten. Furthermore, we used a Generalized Linear Mixed Model (GLMM) to assess our major outcomes. The GLMM predicts a continuous or categorical target base on one or more predictors. GLMM is a particular type of mixed model, and the linear predictor may contain random effects in addition to fixed effects. The random effects are assumed to have a normal distribution, although sometimes the random effect may be excluded. As this is a multilevel model, if the data is missing at level 2, the individual is deleted. However, if the data is missing at level 1, it uses the available data and still estimates the model using that individual’s data. This model allows for nested data structures including longitudinal designs. The main hypotheses used the intent-to-treat approach, including all participants. The GLMM with main effects of treatment (ES and FA) and time, treatment by time interactions and subject-level random intercepts were used to model longitudinal trajectories of the outcomes, employing a log link function for count outcomes. Time was modeled nominally as pre/post. All available observations from each subject were utilized in modeling via the GLMM. Primary outcome models controlled for gender, age, verbal IQ (HAWIVA) and SES. Treatment effect was defined as a significant interaction effect between treatment groups and time. All randomized children were included in all analyses in accordance with intention-to-treat principles.

The moderation effects of treatment with teacher and child relationships were also examined. Teachers’ reports of problem behavior were measured using the *Caregiver-Teacher Report Form –* C-TRF and evaluated using a mixed linear model. The effect of cumulative risk elicited via a guideline-based semi-structured interview with parents (*N* = 122 families), and behavior problems measures using the *SDQ* and attachment was evaluated by analysis of variance (ANOVA). Furthermore, for the impact of psychoanalytic case supervision on the mentalizing capacity of teachers a *t*-test was performed comparing teachers for the ES-group with those of the FA-group.

#### Treatment of Missing Data

Sensitivity tests were performed with various continuous variables to ascertain that data were ‘missing at random’ (MAR). As data were not missing completely at random (MCAR) but certainly at random (MAR), this suggested use of a multiple imputation procedure to avoid data biasing and to maintain statistical power (cf. Graham, 2012). Multiple EM imputation (Rubin, 1996) was implemented using the *Stata mi impute package* to estimate the missing data points.

We have further reduced the potential for biased results by using a large item pool in imputation procedures, which includes variables that may be correlated with missingness, such as baseline aggression and verbal abilities. The cluster effect was accounted for by including an indicator variable. A comparison of background characteristics of the participating sample with those with missing data did not show any significant differences. Missing data included data from both time points. Missing data estimation involved 37.6% of the sample’s main outcome variable (MCAST; 17.1% at baseline prior to intervention and 31.8% from timepoint 2 post intervention). According to Madley-Dowd et al. (2019) these proportions of missing data are acceptable for MI for our MAR data, as a valid MI reduces bias even when the proportion of missingness is large. To capture the random variability around ‘true’ values, the set of missing data points was bootstrap estimated 50 times, thus creating 50 datasets. The results presented here were averaged across datasets using the Stata mi estimate, which utilizes Rubin’s rules CE (Rubin, 1996) for combining results across multiple imputations.

## Results

### Baseline Characteristics

The sample comprised *N* = 526 high-risk children (264 females; mean age 50.83 months), of whom 157 showed a secure attachment classification and 369 an insecure attachment classification (avoidant = 139, ambivalent = 104, disorganized = 126). The sample showed a large percentage of disorganized attachment, with no significant difference between intervention groups at baseline (MCAST – D_*ES*_ 26.3%/D_*FA*_ 22.0%). These percentage rates correspond to a percent rank of 11% in large samples of children at risk from Western countries with low SES (van Ijzendoorn et al., 1999). The sample is characterized by high C-TRF emotional reactivity, aggressive behavior and externalizing problem scores, with no significant difference between intervention groups at baseline (see Supplementary Table S2 for summary of baseline demographic and clinical characteristics).

### Primary Outcome^4^

We report here the change in secure (B) and insecure-disorganized (D) attachment classification of the main outcome criteria at baseline (tpre) and post-intervention (tpost) for the two intervention arms expressed in relative percentage (cf. Table 1); e.g., for B-classifications: the number of children who were classified at tpre as securely attached (B) but not at tpost are compared to those who were classified as insecurely attached (i.e., Non-B) at tpre but as securely attached (B) at tpost^5^. Effect sizes (Cohen’s *d*) were estimated based on the chi-square of change scores and transformed to Cohen’s *d* using the formula by Rosenthal and DiMatteo (2001, p. 72).

**TABLE 1:** Changes of attachment classifications.

Attachment		ES (*N* = 258)	FA (*N* = 268)	Total (*N* = 526)
Baseline tpre	**B** - secure (%)	25.08	34.48	29.87
	*N*	65	92	157
tpost	**B** - secure (%) rel Diff (%)	32.06 6.98	38.21 3.73	35.91 5.32
	Cohen’s d*	0.5459	0.4051	0.4697
	*N* (rel Diff)	83 (18)	102 (10)	185 (28)
Baseline tpre	**D** - disorg. (%)	26.74	22.01	24.33
	*N*	69	59	128
tpost	**D** – disorg. (%) rel Diff (%)	29.84 3.10	25.74 3.73	27.75 3.42
	Cohen’s d*	0.3489	0.4033	0.3729
	*N* (rel Diff)	77 (8)	69 (10)	146 (18)
Attachment		ES (*N* = 258)	FA (*N* = 268)	Total (*N* = 526)
Baseline tpre	B (%)	25.08	34.48	29.87
	*N*	65	92	157
tpost	B rel Diff (%)	6.98	3.73	5.32
	Cohen’s d*	0.5459	0.4051	0.4697
	*N* (rel Diff)	18	10	28
Baseline tpre	D (%)	26.74	22.01	24.33
	*N*	69	59	128
tpost	D rel Diff (%)	3.10	3.73	3.42
	Cohen’s d*	0.3489	0.4033	0.3729
	*N* (rel Diff)	8	10	18

*Transformed Cohen’s *d* based on chi-square (Rosenthal and DiMatteo, 2001, p. 72).

**FIGURE 3:**
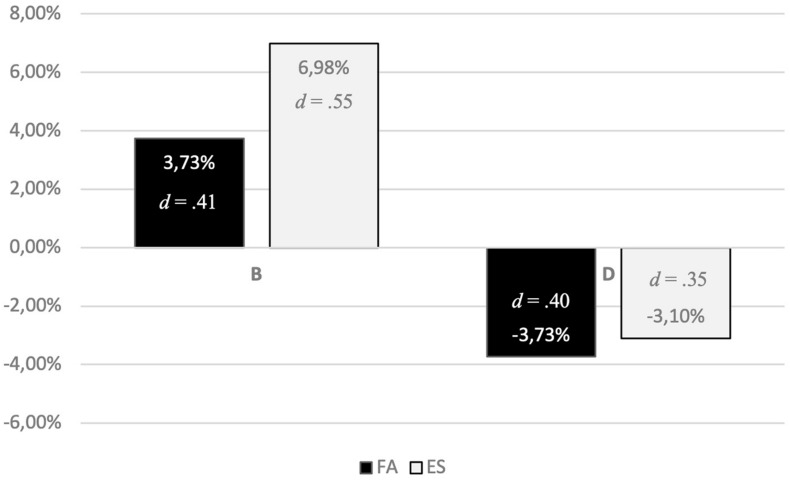
Changes in attachment classification within the 2-year intervention.

Over the 2-year intervention, secure (B) classification in the ES group increased by 6.98%, with a medium effect size of *d* = 0.5459, whereas the secure (B) classifications in the FA group increased by 3.73%, with a small-medium effect size of *d* = 0.4051. Disorganized (D) classification declined by 3.1% in the ES-intervention group and by 3.73% in the FA group, both having small-medium effect sizes (*d* = 0.3489/0.4033; see Figure 3 and Table 1). The overall effect size for the total sample is medium for changes in a secure classification (*d* = 0.4697) and small-medium for changes away from a D classification (*d* = 0.3729; Cohen, 1988).

**FIGURE 4:**
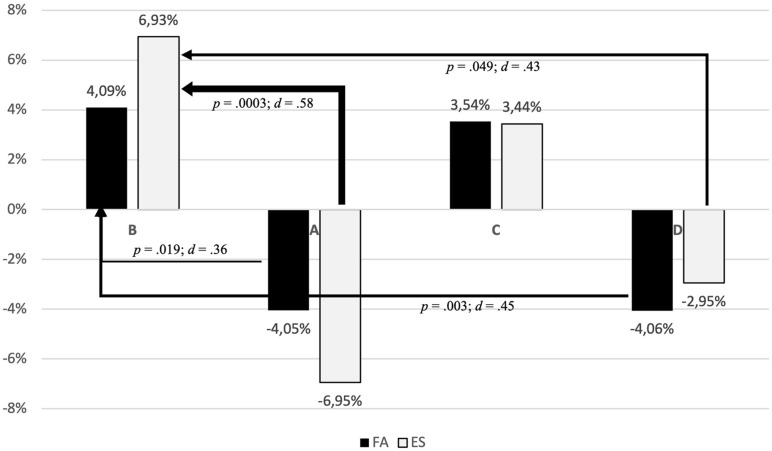
Detailed shift of per-post attachment classification.

Both intervention groups (ES and FA) showed a significant shift from D to B classification, with an intermediate effect size (*d*_*ES*_ = 0.43; *d*_*FA*_ = 0.45). Furthermore, both intervention groups showed a significant change from A to B classification, with a medium effect size for the ES group (*d*_*ES*_ = 0.58) and only a small effect size (*d*_*FA*_ = 0.36) for the FA group (see Figure 4).

*Attachment classification – MCAST in a group comparison of ES vs. FA.* In a second analysis, we compared the effect on attachment classifications in the two intervention arms – i.e., which intervention changed attachment classification more effectively? Group comparisons of children with secure vs. non-secure attachments showed a significant change of MCAST classification over time by treatment group (*t* = −3.277, *p* = 0.001), with an effect size of Cohen’s *d* of 0.142 (see Table 2). The exp coefficient of 0.773, which is the odds ratio (OR), favors the intervention group ES, where the odds of becoming classified as B post-intervention increases by this factor in comparison to FA. The effect of Cohen’s *d* of 0.142 for the comparison of treatment groups by time may be interpreted as small.

**TABLE 2:** Estimated coefficients of the generalized linear mixed model of the primary outcome MCAST (attachment).

	B - secure vs. non-B - insecure						
*Model term*	*Coeff.*	*SE*	*t*	*Sig.*	*Exp coeff.*	*95% Conf. Int.*	*d*
Trial*Group	–0.257	0.0784	–3.277	**	0.773	0.663 – 0.902	0.142
Trial*Group*Sex	1.039	0.1083	9.596	***	2.827	2.287 – 3.496	0.5729
D vs. non-D							
Trial*Group	1.316	0.1033	12.740	***	3.729	3.046 – 4.566	0.7258
Trial*Group*Sex	–1.519	0.1515	–10.024	***	0.219	0.163 – 0.295	0.8373

**p ≤ 0.01; ***p ≤ 0.00.

Trial by treatment by gender interactions indicated that the effect of treatment varied by gender, favoring ES-group girls (*t* = 9.596, *p* < 0.0001; OR: 2.827; Cohen’s *d*: 0.5729; see Table 2), having an intermediate effect on girls becoming more securely attached after ES intervention.

The proportion of disorganized MCAST classifications also decreased by treatment group over time (*t* = 12.740, *p* = 0.0001; OR = 3.729; Cohen’s *d*: 0.7258; see Table 2), indicating that the ES group had significantly more change toward organized attachment classifications post-intervention, having an intermediate-large effect size (Cohen, 1988). Trial by treatment by gender interactions indicated a slight advantage favoring FA-group boys (*t* = −10.024, *p* < 0.0001; OR: 0.219; Cohen’s *d*: 0.8373; see Table 2), having a large effect on boys becoming more organized-attached after FA intervention compared to boys in the ES intervention.

### Secondary Outcome for the Central Research Question

#### (a) Caregiver-Teachers Evaluation on Child Behavior

*Caregiver-Teacher Report Form.* In the overall sample, problem behavior significantly improved from baseline to the end of treatment (coefficient: −5.91; *t* = −4.67, *p* < 0.001). A significant treatment effect was found in which the FA group improved more than the ES group (coefficient: 4.45; *t* = 2.49, *p* = 0.013), particularly in the domains of aggressive behavior (coefficient: 2.49; *t* = 3.54, *p* < 0.001) and externalizing problems (coefficient: 3.04; *t* = 3.28, *p* = 0.001).

#### (b) Risk-Factors and Resilience^6^

In a sub-study of “EVA” by Neubert (2016) the specific living circumstances of the participating at-risk-children (low SES, rural areas with high unemployment, migration background) were addressed. The sub-study examined specific risk factors the children were facing and their effects on children’s behavior problems. The main findings refer to cumulative interaction of risks as well as influences of single risk factors on the severity of children’s behavioral disorders. Additionally, the effect of a secure attachment representation under conditions of cumulative risk on the development of behavior problems was tested.

The data on risk exposure of the families and children’s problematic behavior was assessed by conducting a guideline-based semi-structured interview with the parents asking them about their families living circumstances, e.g., single-parenthood, divorce, loss, trauma, migration history, incidents during pregnancy/early childhood, illness, parental stress, education, income and unemployment. Attachment representation was assessed by the MCAST (s.a) and behavioral problems were obtained with the help of the total difficulties score of the SDQ. The sub-study included a sample of *N* = 122 families who took part in the “EVA”-study.

##### Cumulative risk and behavior problems

**FIGURE 5:**
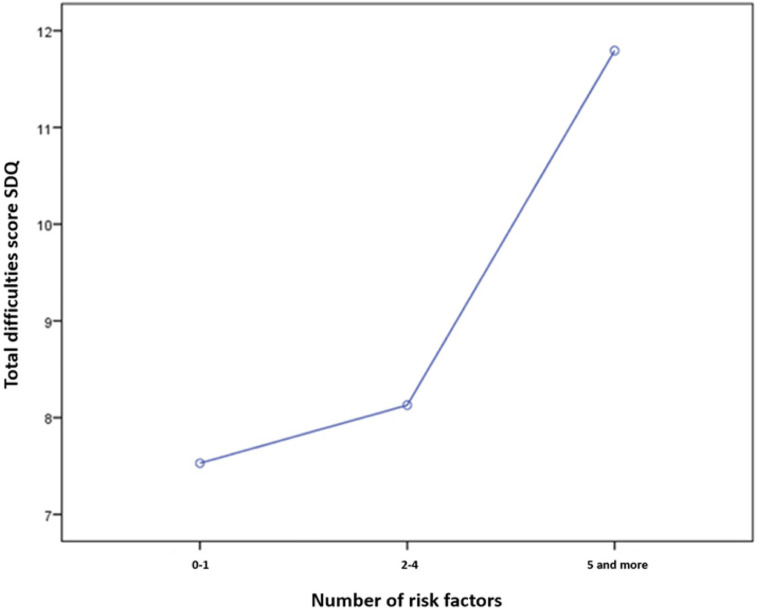
Number of risk factors in relation to total difficulties score of the SDQ.

An analysis of variance showed a statistically significant correlation between the number of risk factors and the amount of children’s behavior problems: The more risk factors the children were facing the higher was their SDQ total difficulties score [*F*_(__2_,_119__)_ = 7.234, *p* = 0.001, η^2^ = 0.108; see Figure 5].

Regarding the relation of risk factors with the occurrence of problematic behavior (via spearman rank test), psycho-social or family-related risk factors appeared to be of high relevance (*r* = 0.420, *p* = 0.01) whereas class-related or socio-economic factors did not show to be directly related to the SDQ total difficulties score (*r* = 0.011, *n.s.*).

Single risk factors which proved to be most important, due to a regression analysis (*R*^2^ = 0.213), were parental stress level (β = 0.298, *p* < 0.001), the experience of violence within the family (β = 0.184, *p* < 0.05) or traumatic events within the family (β = 0.209, *p* < 0.05).

##### Cumulative risk, attachment and behavior

**FIGURE 6:**
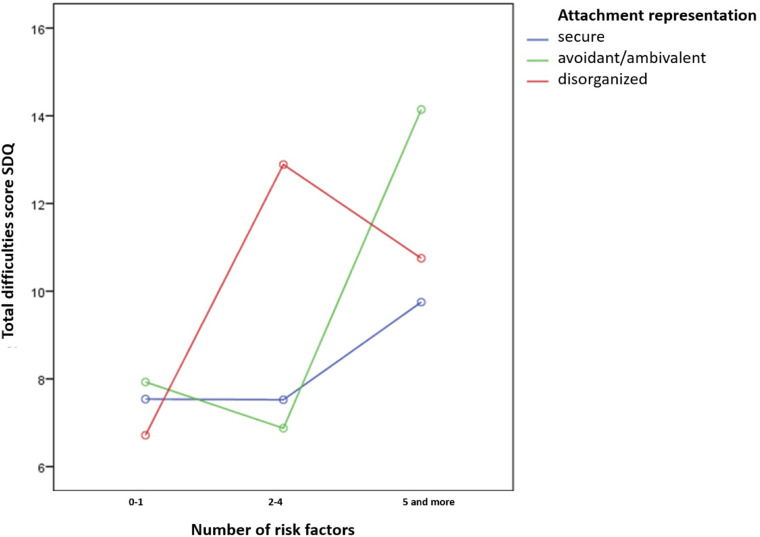
Attachment representations in relation to number of factors and total difficulties score–SDQ.

A further research interest was to explore to which extend secure attachment representation acts as a protective factor for developing behavioral disorders as a function of risk. The analysis of variance (ANOVA) revealed an interaction of the attachment representation and the number of risk factors in regard to the severity of problematic behavior [*F*_(__4_,_113__)_ = 3.374, *p* = 0.012, η^2^ = 0.107; see Figure 6].

Due to our analysis of simple main effects, there was no significant difference between the three attachment groups regarding the amount of behavior problems on risk level 1 (0–1 risk factors). Under the condition of moderate risk (2–4 risk factors) the disorganized children showed a significant increase in the amount of problem behavior (*p* = 0.014^∗^). In the case of more than five risk factors (risk level 3) also the insecure avoidant/ambivalent group showed a significant increase in the total difficulties score of the SDQ (*p* = 0.001^∗^) for comparing risk level 1 versus risk level 3, *p* = 0.000^∗^ for comparing risk level 2 versus risk level 3 (^∗^*p* < 0.05). The decrease of the total difficulties scores in the disorganized group from risk level 2 versus risk level 3 was not significant (0.373, *n.s*.). The findings indicate that a secure attachment representation acts as a protective factor by preventing children from developing behavioral problems, especially when they are facing a high-risk environment. This is in line with the theoretical approach of “EVA,” which is to affect the attachment representation from a disorganized or insecure into a more secure direction to prevent children’s behavioral problems.

#### (c) Capacity of Self-Reflection and Mentalization of Teachers and Their Influence on Supporting Children-at-Risk^7^

The main aim of this analysis was to examine the impact of psychoanalytic case supervision on the mentalizing capacity of teachers in the EVA study. Especially focusing on possible differences between the two teacher groups in FA and ES respectively, based on the hypothesis that the ES group might gain higher Reflective Self Ratings than the FA group due to the received psychoanalytic case supervision.

**FIGURE 7:**
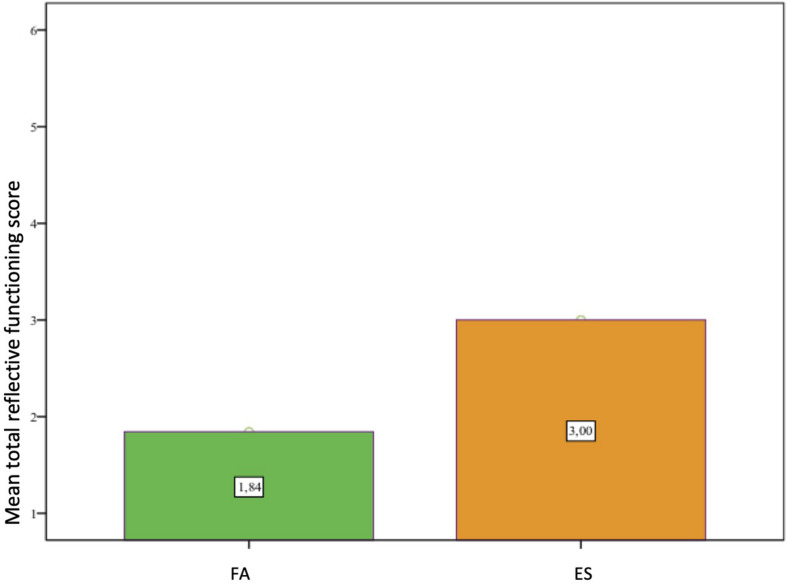
Reflective Functioning Scores of teachers in FA- and ES- group. FA: *N* = 32; *m* = 1,84; *sd* = 1,32. ES: *N* = 32, *m* = 3,00; *sd* = 1,54.

A *t-*test of the rated IPR interview with all teachers (*N* = 64) of both groups showed a statistically significant difference between the two groups. The ES group, that had received the case supervision, achieved on average higher Reflective Functioning Scores than the FA-group [*t*(60) = −3,22, *p* = 0.002; *d* = 0.82; see Figure 7].

A regression analysis confirmed that the group membership, i.e., receiving supervision, explained the group difference of the mentalization capacity [DV = “Reflective Functioning” (β = 0.378, *p* = 0.005)]. The group membership can therefore be seen as a significant predictor of the Reflexivity (*N* = 64; *R*^2^ = 0.143). In addition, the qualitative analysis of the interviews showed clearly that the supervision was perceived by teachers as enhancing their professional development (Asseburg, prev. Hartmann et al., 2015).

**FIGURE 8:**
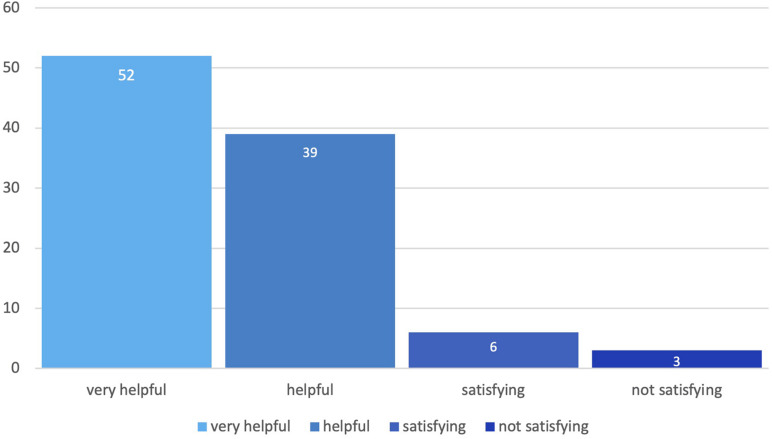
Perceived benefit of the supervision.

Figure 8 shows the results of the qualitative data analysis of the remarked supervision benefit by the interviewed teachers. 52% perceived the supervision as “very helpful,” 39% as “helpful,” 6% as “satisfying” and 3% as “not satisfying” (*N* = 31).

These results provide the first evidence for the positive enhancing influence of psychoanalytic case supervision on the mentalizing capacity of early pedagogical teacher and can be seen as a very crucial variable in the process of the early prevention of the EVA Study having in mind that teachers play an important role in the development of children, potentially offering a “secure base experience” and enhancing epistemic trust. In particular when they are enabled to offer the child a stable emotional relationship, which is greatly aided by mentalizing skills.

## Discussion

The drifting apart of the developmental chances of children who are better supported in today’s Western societies than in any previous historical period and those who live on the fringes of society has continuously increased in the last decades (see e.g., Emde and Leuzinger-Bohleber(eds), 2014). This promotes processes of division and even splitting in society, which many see as a serious threat to Western democracies. In the Corona crisis these processes could be clearly observed in a dramatic way: as many media reports described, it were again the children from socially disadvantaged families who suffered most from the lock-down, both in terms of their educational opportunities and the risk of being exposed to domestic violence.

To meet this threat more and more professional groups are getting involved in early prevention, so that today we have a wide range of early prevention programs. Unfortunately, only a few have been carefully scientifically evaluated and examined the short and long-term effects of prevention on children-at-risk.

The EVA study aimed specifically at comparing such short- and long-term outcomes of two early prevention offers in a large sample of Kindergarten-aged children at high psychosocial risk, one (ES) being a psychodynamic, attachment-based holistic approach, the other (FA), a classroom-based, psychodynamically inspired prevention.

Baseline data showed that the children examined in EVA can indeed be considered as “children-at-risk”: They had twice as much disorganized attachment representations than those of a normal population (ES: 26.3%; FA: 22%; normal population approx. 11%) which is considered as an indicator that these children had gone through severe early trauma (often in the context of domestic violence) running a serious developmental risk (see Emde and Leuzinger-Bohleber(eds), 2014). These children have not been able to develop stable and sustaining inner working models but rather showed “disorganized attachment patterns,” i.e., they did not develop sufficiently an inner feeling of safety, of confidence in the capability of the self to cope with difficult situations in a creative way and did not find reliable human objects to help them in situations of emergency. From a psychodynamic point of view this is one reason why these children react with panic, aggression and violence or even with a decompensation of their psychic stability in personal and social stress situations (see e.g., Leuzinger-Bohleber, 2010). Therefore, many professionals and researchers have claimed that children exhibiting disorganized attachment representations should receive psychological and psychosocial support as early as possible. The EVA study pursued this ambitious goal although there had been serious doubts if these children-at-risk and their families could be reached by the psychodynamically oriented offers in public Kindergartens.

It therefore is a first important finding that the results of the EVA study show that indeed both prevention offers affected these children-at-risk: The disorganized attachment representation (D) decreased in the ES group by 3.1% (*d* = 0.35), in the FA group by 3.73% (*d* = 0.40). Both prevention groups lead to a significant shift from a disorganized attachment representation (D) to a secure attachment representation (B; ES: *d* = 0.43; FA: *d* = 0.45). In the ES groups the disorganized attachment representation decreased more intensively than in the FA group (*d* = 0.73). Against the background of many long-term studies in the field of attachment research (see e.g., Fonagy and Campbell, 2015), this is an important finding, because it justifies the hope that children who have developed a disorganized attachment due to traumatic early experiences in their first year of life can indeed benefit from prevention programs in public Kindergartens and even ameliorate their problematic attachment. These results have important clinical and social relevance, as disorganized attachment representation is a known risk marker for later externalizing disorder and social difficulty (Fearon et al., 2010; Groh et al., 2012, 2014; Helmsen, et al., 2012; Granqvist et al., 2017).

Therefore, the most important result of the EVA study is that both interventions effectively ameliorated the attachment representations in children: In the ES group the secure attachment representation (B) increased by 6.98% (*d* = 0.55), in the FA group by 3.73% (*d* = 0.41). There also was a significant shift from an avoidant attachment representation (A) to a secure attachment representation (B; ES: *d* = 0.58; FA: *d* = 0.36). Concerning the *secondary outcome measure* C-TRF: according to their teacher’s evaluations, children’s behavioral problems decreased in both prevention groups (*p* < 0.001), where children in the FA group improved particularly in aggressive behavior (*p* < 0.001).

Changes in attachment representations thus have a positive effect on social behavior, but assumable also on cognitive and affective development. Especially the interaction of modules that focus on the specific, idiosyncratic traumatization of the individual child (such as child therapies and through case supervision) and those that benefit the entire “Kindergarten” system (team supervision, advanced training, *Faustlos*, parental work) has proven its worth. All these changes can be seen as indicators that the resiliency of these children-at-risk has been improved by the early prevention (see also Hauser et al., 2006; Leuzinger-Bohleber, 2009; Cicchetti, 2010; Goldstein and Brooks, 2013).

In addition to the main outcomes of the EVA study the results of two sub-studies have been summarized in this paper. Neubert (2016) addressed the specific living circumstances of the participating families and examined their effects on children’s behavioral problems. Parental stress level and the experience of violence or trauma within the family appeared to be the most important single risk factors for developing problem behavior. There was also a statistically significant correlation between the number of risk factors and the severity of children’s behavioral disorders: the more risk factors the children were facing, the higher was their SDQ total difficulties score. The main finding refers to the interaction of attachment representation and the number of risk factors in regard to the severity of problematic behavior: The findings indicate that a secure attachment representation acts as a protective factor by preventing children from developing behavioral problems, especially when they are facing a high-risk environment. Based on these findings, the authors argue for the conception of individual prevention and support offers which consider the specific living conditions of children and also have a relationship-based approach.

A second sub-study by Asseburg (prev. Hartmann et al., 2015) examined the impact of psychoanalytic case supervision on the mentalizing capacity of early educational professionals (teachers) in the EVA study. It revealed that teachers’ self-reflective capacities only increased in the ES group. It became apparent that self-reflective skills of teachers are essential to support children-at-risk in a highly individual way in order to strengthen their resilience. The improvement from problematic to less problematic attachment patterns in this high-risk sample is seen as a result of the teachers’ increased vocational competencies.

### Limitations

Like every study, the EVA project has its limitations. One of the limitations of this study is that we could not analyze the mediator and moderator variables in detail. The results of the two sub-studies indicate that the two prevention programs ES and FA have differential effects, but these need to be investigated further. Thus, although our two main questions about these differential effects of the two prevention programs could be answered globally, the mediator and facilitator effects should be empirically investigated in detail. Observations suggest that on the children’s side, the following mediators should be studied: severity of the disorder, resilience factors, traumatization of the parents, willingness of the parents to cooperate in early prevention, other family risk factors (poverty, divorce of the parents, unemployment, attachment type). As with respect to the institutions we assume the following mediators: Childcare ratio of teacher/children, professional experience of the teachers, team factors (collegiality, mutual support, atmosphere etc.), dealing with situations of excessive demands (indicators: number of sick leaves of the Kindergarten teachers, participation in supervision, further trainings, attachment type of teachers compared to those of the children or other teachers etc.). The influence of such factors could be the subject of further studies.

Additionally, the finding that a prevention program such as FA, focusing mainly on social learning processes in groups, might also help disorganized children (here especially boys) change their problematic attachment patterns toward more organized ones also is an interesting, unexpected outcome, which should be further studied in the future.

### Implications for Research, Policy and Practice

From a societal perspective, the results of the EVA study are relevant: The development of children-at-risk growing up in deprived social environments can be effectively supported by early prevention programs that use the local professional networks for prevention in socially burdened urban environments. The additional financial costs of ES compared to FA is worthwhile insofar as the attachment representation of children-at-risk can be changed for the better, which sustainably increases the developmental chances of these vulnerable children. The study also shows that experienced psychotherapists can successfully apply their professional knowledge to children at risk in their natural environment and thus counteract the increasing schism between social groups. It is also worth mentioning that the psychodynamic psychotherapists have further professionalized their competence due to their experiences in the prevention projects over the years, which even has, among other things, positive effects on their training.

During the time of the EVA studies, most child therapists in Frankfurt a. M. were psychodynamically oriented. Therefore, it was a request of some of the political leaders in the field of early education in this city to systematically use the professionalism of these therapists to support children with poor developmental chances due to an accumulation of social and psychological risk factors in certain areas of the city. The Frankfurt Prevention Study as well as the EVA Study show that child psychotherapist can certainly fulfill the expectations placed in them by politicians. Other studies could show that this is also the case with child therapists trained in other therapeutic schools. As Heckmann (2008) has shown, early prevention is not only a sustainable benefit for the psychological and psychosocial development of children at risk, but also saves enormous costs in the long run.

## Data Availability Statement

The raw data supporting the conclusions of this article will be made available by the authors, without undue reservation.

## Ethics Statement

The studies involving human participants were reviewed and approved by LPPKJP – Landeskammer für psychologische Psychotherapeutinnen und -therapeuten und Kinder- und Jugendlichenpsychotherapeutinnen und -therapeuten Hessen. Written informed consent to participate in this study was provided by the participants’ legal guardian/next of kin. Written informed consent was obtained from the minor(s)’ legal guardian/next of kin for the publication of any potentially identifiable images or data included in this article.

## Author Contributions

ML-B was the PI of the EVA Study. ML-B conceptualized this manuscript and wrote a first draft in close cooperation with TF. TF collected the data and analyzed the data together with FH. FH helped in recruitment, analysis and writing as part of her doctoral thesis in clinical psychology. VN and LA conducted and evaluated the substudies within EVA and wrote their doctoral thesis on the subject. JG and MW supervised all stages of the workincluding conceptualization, analysis, and writing. TF and ML-B are jointly accountable for the content of the work, ensuring that all aspects related to accuracy or integrity of the study are investigated and resolved in an appropriate way. All authors contributed to the article and approved the submitted version.

## Conflict of Interest

The authors declare that the research was conducted in the absence of any commercial or financial relationships that could be construed as a potential conflict of interest.

## Footnotes

1 See also Section “Preventions.”

2 Formula: = {1 + [(1 + *CV*^2^)m − 1] *ICC*} *n.*

3 http://www.cfchildren.org/second-step

4 We are planning to publish the following Section “Randomization, Sample Size and Methods to Preserve Blindness”) in a modified version in another paper with a different focus of content and argumentation.

5 Relative differences in attachment classification at tpost were calculated from baseline by dividing the squared difference of the attachment classifications from tpre to tpost by the sum of the two (*d*_*pre,post*_ = (*n*_*pre,post*_ − *n*_*post,pre*_)^2^/*n*_*pre,post*_ + *n*_*post,pre*_).

6 Neubert, Verena: doctoral thesis (2016). Substudy of EVA including a subsample of *N* = 122 families.

7 Doctoral thesis Lorena Asseburg (prev. Hartmann) 2015.
