# Supplementary material for: Can Psychodynamically Oriented Early Prevention for “Children-at-Risk” in Urban Areas With High Social Problem Density Strengthen Their Developmental Potential? A Cluster Randomized Trial of Two Kindergarten-Based Prevention Programs
Source: Front Psychol. 2020 Dec 10;11:599477. doi: 10.3389/fpsyg.2020.599477 (PMC7759147; doi:10.3389/fpsyg.2020.599477)
Supplement: Supplementary file 1 [file Table_1.DOCX]

Table S1. Clustering by SES high/low, hyperactivity in children high/low, aggressive behavior in children high/low and Kindergartens contacted to be screened for eligibility and to be randomized for the EVA-Study thereafter

|  | **SES low**^1^ | | **SES high**^2^ | |
| --- | --- | --- | --- | --- |
|  | Cluster Nbr (N) | screened Kindergartens (N) | Cluster Nbr (N) | screened Kindergartens (N) |
| **Hyperact. high Aggressiv. high** | 1 (12) | 6 | 2 (10) | -- |
| **Hyperact. high Aggressiv. low** | 3 (12) | 1 | 4 (9) | -- |
| **Hyperact. low Aggressiv. high** | 5 (5) | 3 | 6 (14) | 1 |
| **Hyperact. low Aggressiv. low** | 7 (14) | -- | 8 (10) | -- |
| **Hyperact. unknown Aggressiv. unknown** | 9 (12) | 4 | 10 (16) | 1 |

^1^ SES low: urban area with Germans with Migration background 14.6%; Foreign nationalities 27.4%; Unemployment 7.5%.

^2^ SES high: urban area with Germans with Migration background 12.4%; Foreign nationalities 19%; Unemployment 5.7%.

Source: Inhabitants with main dwelling in Frankfurt am Main on 31. December 2008 according to neighborhoods, Germans with and without migration background in %, foreign nationalities in %: statistical yearbook Frankfurt am Main 2009, p. 36; unemployment density in Frankfurt am Main on June 2008; statistical yearbook Frankfurt am Main 2009, p.107
