# Supplementary material for: Can Psychodynamically Oriented Early Prevention for “Children-at-Risk” in Urban Areas With High Social Problem Density Strengthen Their Developmental Potential? A Cluster Randomized Trial of Two Kindergarten-Based Prevention Programs
Source: Front Psychol. 2020 Dec 10;11:599477. doi: 10.3389/fpsyg.2020.599477 (PMC7759147; doi:10.3389/fpsyg.2020.599477)
Supplement: Supplementary file 2 [file Table_2.docx]

Table S2. Sociodemographic characteristics and baseline measures by intervention group

|  | Treatment | | | | | | | | | | | | | | | | | | | | | |
| --- | --- | --- | --- | --- | --- | --- | --- | --- | --- | --- | --- | --- | --- | --- | --- | --- | --- | --- | --- | --- | --- | --- |
|  | ES (*n* = 258) | | | | | | | | | |  | FA (*n* = 268) | | | | | | | | | | |
|  | | *n* (%) | | | *M* (*SD*) | | | Range | | |  | *n* (%) | | | | *M* (*SD*) | | | | Range | | |
| **Family** |  | |  | | | | | | |  |  |  | | |  | | | | | | |  |
| Low SES^a^ | 212 (82.2%) | | | | |  | | | |  |  | 253 (94.4%) | | | | | |  | | | |  |
|  |  | |  | | | | | | |  |  |  | | | | |  | | | | |  |
| **Children^b^** |  | |  | | | | | | |  |  |  | | | | |  | | | | |  |
| Age (months) | |  | 50.66 (8.585) | | | | 24.0-68.0 | | | |  |  | | 51.0 (10.565) | | | | | 26.0-68.0 | | | |
| Sex |  | |  | | | | | | |  |  |  | | | | |  | | | | |  |
| Girl | 123 (47.7%) | | | | |  | | | |  |  | 141 (52.6%) | | | | | |  | | | |  |
| Boy | 135 (52.3%) | | | | |  | | | |  |  | 127 (47.4%) | | | | | |  | | | |  |
|  |  | |  | | | | | | |  |  |  | | | | |  | | | | |  |
| **Verbal IQ** |  | | | 89.84 (17.328) | | | | |  | |  |  | | 91.19 (14.897) | | | | | |  | | |
|  |  | |  | | | | | | |  |  |  | | | | |  | | | | |  |
| **Attachment classification**^c^ | | | | | |  | | | |  |  |  | | | | |  | | | | |  |
| disorg (D) | 68 (26.3%) | | | | |  | | | |  |  | 58 (22.0%) | | | | |  | | | | |  |
| ambiv (C) | 49 (19.0%) | | | | |  | | | |  |  | 55 (20.2%) | | | | |  | | | | |  |
| avoid (A) | 76 (29.4%) | | | | |  | | | |  |  | 63 (23.1%) | | | | |  | | | | |  |
| secure (B) | 65 (25.2%) | | | | |  | | | |  |  | 92 (34.7%) | | | | |  | | | | |  |
|  |  | |  | | | | | | |  |  |  | | | | |  | | | | |  |
| **CTRF**^d^ |  | |  | | | | | | |  |  |  | | | | |  | | | | |  |
| em_react |  | | 1.37 (1.911) | | | |  | | | |  |  | | | 1.38 (1.769) | | | | | |  | |
| anx_depr |  | | 2.75 (2.822) | | | |  | | | |  |  | | | 2.66 (2.549) | | | | | |  | |
| soc_withdr | |  | 2.81 (3.290) | | | |  | | | |  |  | | | 2.75 (2.897) | | | | | |  | |
| att_deficit |  | | 3.88 (4.191) | | | |  | | | |  |  | | | 3.84 (3.962) | | | | | |  | |
| aggressive |  | | 7.05 (8.599) | | | |  | | | |  |  | | | 7.57 (9.085) | | | | | |  | |
| int_probl |  | | 7.93 (7.484) | | | |  | | | |  |  | | | 7.80 (6.887) | | | | | |  | |
| ext_probl |  | | 10.91 (12.108) | | | |  | | | |  |  | 11.41 (12.265) | | | | | | | |  | |

*Note* disorg – insecure-disorganized; ambiv – insecure-ambivalent; avoid – insecure-avoidant; em_react – emotional reactivity; anx_depr – anxious/depressive; soc_withdr – social withdrawal; att_deficit – attention deficit; aggressive – aggressiveness; int_probl – internalizing problems; ext_probl – externalizing problems.

^a^ FA family SES was significantly lower from SES in ES Families, *U* = 30343.0, *p* = .000, *r* = .19

^b^ No significant differences by treatment group in age *t(509)* = .398, *p* = .691, gender *U* = 32865.0,
*p* = .258, *r* = .05 or verbal IQ *t*(353) = .803, *p* = .422

^c^ No significant differences by treatment group, *𝜒* ^2^= 6.8113, *p* = .078162

^d^ No significant differences by treatment group in CTRF em_react *t(498)* = .079, *p* = .937; anx_depr *t(498)* = -.345, *p* = .730; soc_withdr *t(498)* = -.231, *p* = .817; att_deficit *t(498)* = -.126, *p* = .900;
aggressive *t(498)* = .658, *p* = .511; int_probl *t(498)* = -.201, *p* = .841; ext_probl *t(497)* = .455, *p* = .649
