# Supplementary material for: Can Psychodynamically Oriented Early Prevention for “Children-at-Risk” in Urban Areas With High Social Problem Density Strengthen Their Developmental Potential? A Cluster Randomized Trial of Two Kindergarten-Based Prevention Programs
Source: Front Psychol. 2020 Dec 10;11:599477. doi: 10.3389/fpsyg.2020.599477 (PMC7759147; doi:10.3389/fpsyg.2020.599477)
Supplement: Supplementary file 3 [file Data_Sheet_1.docx]

*Clustering details*

Clusters were kindergartens from neighborhoods known as deprived with respect to SES and high migration population within the City of Frankfurt, chosen from a total sample of 114 kindergartens with over 4500 children, where more than 2700 children were within the age range of three to four years. A representative sample for the EVA study was drawn on the basis of SES, migration background, and child problem behavior and socio-emotional competency, as evaluated with the ‘Verhaltensbeobachtungsbogen’ questionnaire (VBV [behavior observation questionnaire]; Döpfner et al., 2001). Clusters were built taking the social structure of the urban areas of the kindergartens as well as the number of psychologically conspicuous children per kindergarten (oppositional-aggressive behavior, hyperactivity) into account. Kindergartens were randomly picked primarily from low SES urban areas then contacted asking their willingness to participate and after consent to participate Kindergartens were randomized to one of the intervention arms (see Table S1 available online).

**References**

1. Döpfner, M., Berner, W., Fleischmann, T. & Schmidt, M. H. (2001). Verhaltensbeurteilungsbogen für Vorschulkinder (VBV). Weinheim: Beltz Test
